# Water Regime Monitoring of the Royal Walnut (*Juglans regia* L.) Using Sap Flow and Dendrometric Measurements

**DOI:** 10.3390/plants10112354

**Published:** 2021-10-30

**Authors:** Viliam Bárek, Martina Kováčová, Vladimír Kišš, Oleg Paulen

**Affiliations:** 1 Department of Water Resources and Environmental Engineering, Faculty of Horticulture and Landscape Engineering, Slovak University of Agriculture, Tr. A. Hlinku 2, 949 76 Nitra, Slovakia; viliam.barek@uniag.sk; 2 Research Centre Agrobiotech, Slovak University of Agriculture, Tr. A. Hlinku 2, 949 76 Nitra, Slovakia; vladimir.kiss@uniag.sk; 3 Faculty of Horticulture and Landscape Engineering, Institute of Horticulture, Slovak University of Agriculture, Tr. A. Hlinku 2, 949 76 Nitra, Slovakia; oleg.paulen@uniag.sk

**Keywords:** walnut, sap flow, irrigation, budbreak, flowering

## Abstract

Changes in the distribution of annual rainfall totals, together with the increase in temperature over the last 40 years, are causing more frequent periods of drought, and plants are more often exposed to water stress. The aim of this study was to monitor the effect of different water regimes (irrigated and non-irrigated) of individuals of walnut tree (*Juglans regia* L.) in a private orchard located in the West of Slovakia. Our research was focused on dendrometric and sap flow measurements in the period from 28 March to 2 June 2019. The results showed differences in the sap flow of walnut trees during the budbreak period: when trees were irrigated, sap flow in the diurnal cycle was around 130 g·h^−1^ (20.48%), higher than in the non-irrigated treatment. Dendrometric differences between the irrigated and non-irrigated treatments were not significant. The sap flow data in the flowering period of the irrigated variant were slightly higher at 150 g·h^−1^ (35.62%) than non-irrigated. Dendrometric differences were more significant when the difference between the variants was more than 1.5 mm. Continuation of this research and analysis of the data obtained in the coming years will allow us to evaluate the effects of the environment on fruit trees in the long term.

## 1. Introduction

Climate change affects all sectors of human activities, including agriculture [1]. Brázdil et al. [2] expect an increase in the air temperature in Central Europe of 1.2–1.5 °C by 2050 and 3.3 °C by 2100. As part of climate change, it is expected that the precipitation regime will change, and agricultural crops will be exposed to various forms of biotic and abiotic stress in the coming years [3,4]. Changes in the distribution of annual rainfall totals, together with the increase in temperature over the last 40 years, are causing more and longer periods of drought [5,6,7], and plants are more often exposed to water stress.

Walnut is a worldwide cultivated hardwood fruit species. The production of walnuts in 2019/2020 was approximately 2.1 million metric tons [8]. Walnut is a rich source of unsaturated fatty acids, vitamins and nutrition that can substitute animal protein source [9].

Water stress is a strong limiting factor that can reduce plant growth [10,11]. Trees and woody plants can change their cell wall thickness or the length and diameter of xylem channels in response to persistent drought [12]. Water deficit has a significant impact on the production of walnut trees, not only in terms of the nut yield but also its quality [13,14,15]. Although walnut roots grow to a depth of 2.5 m, a significant part of the fruit declines in dry conditions; the fruits have thinner pericarp; and the leaves are narrower, twisted, yellow, and sometimes fall prematurely [16].

Various methods have been developed over the years to monitor plants during periods of water stress. The heat balance measurement methods measure the water flow in the plants [17]. These methods are typical by their versatility as they can be applied for any plant and trees species, as well as on any plant body parts. They are primarily divided into invasive methods and non-invasive methods. In the case of invasive methods, the heat probes are implanted in the plant part and can cause damage of the plant tissue. In non-invasive methods, the probes are placed in close contact with the plant part [18].

The measurement of dendrometric changes in the plant stems or branches is another widespread method of water stress estimation. The development of the water supply in the trunk can be determined based on the intensity of expansion and contraction of wood and bark during the year. The long-term increase in diameter is reflected as the irreversible increment of the biomass of the bark; on the other hand, short-time swelling is caused by temporarily storing water in the tissue under the bark. With the use of high-frequency data recording with electric dendrometers, it is possible to determine the timing and total phase length of the intensive thickness increment as well as the total annual increment with high accuracy. A negative growth increment (strain shrinkage) in the growing season is the result of soil moisture deficit, while natural strain shrinkage is observed when frosts occur [19,20]. The physiological changes in plant tissues depend on many factors such as meteorological conditions, growth stage, soil type, soil moisture, or susceptibility of plant species to drought [21,22,23]. 

The aim of this study was to monitor the effect of weather changes on the water regime of walnut tree (*Juglans regia* L.) in an orchard located in southwestern Slovakia by the heat balance method using non-invasive surface sensors to monitor sap flow. We hypothesized that sap flow rate and radial branch increment are higher in trees of irrigated treatment than in trees of non-irrigated treatment. In Section 2—Materials and Methods, we describe the background of the experiment and the methods used to measure and collect data. This chapter is divided into three sub- chapters (2.1. Experimental site, 2.2. Experimental design, and 2.3. Field measurements). Sub- chapter 2.3. contains the description of all measuring methods (soil moisture, sap flow and dendrometric measurements, and statistical analyses). Section 3—Results is divided into three sub-chapters. These sub-chapters describe the results of data collected in the budbreak period (3.1.) and flowering period (3.2.) as well as the relationships between sap flow rate and other measured values.

## 2. Materials and Methods

Our study was performed in the field conditions of a fruit orchard to collect real measured data of air temperature, rainfall, and soil moisture in three different depths. Other measured data were sap flow and the dendrometric changes of branch diameter. Data were collected in 1-hour intervals throughout the experiment in irrigated and non-irrigated treatments.

### 2.1. Experimental Site

**Figure 1:**
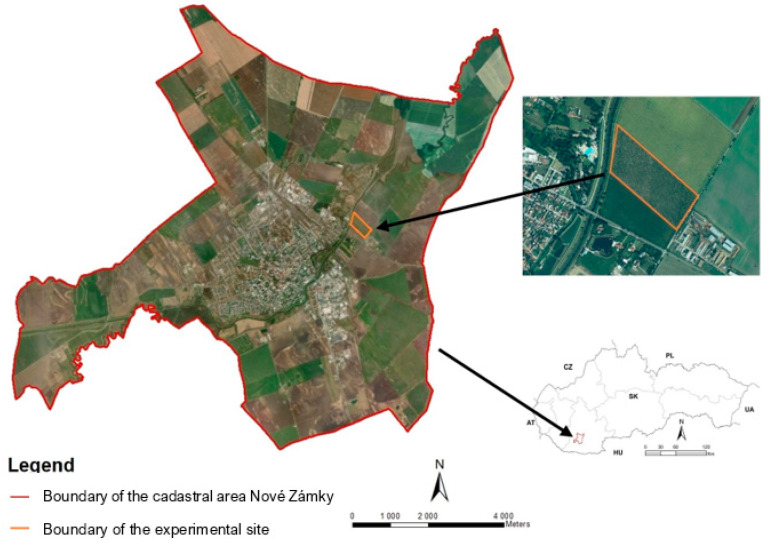
Location of the experimental site.

The experiment was established at a private walnut orchard. The site is located near the town of Nové Zámky (47°59′49″ N; 18°11′26″ E) (Figure 1) with an altitude of 115 m above sea level. The groundwater level in this area is 3 m. The slope of the experimental site is 0°. This area is included in the geological unit of Danubian Hills, subunit Nitra Meadow (Nitrianska niva), part of the Novozámocká plain (Novozámocká pláňava). The site is dominated by cultivated chernozems, mostly calcaric. According to the results of pedological analysis by the Slovak University of Agriculture in Nitra in 2017, the soil type in this area is represented by loamy soils, and in a depth of 0.40 to 0.50 m is the layer of clay loam soil type. The content of organic content in the substrate is 1.61%. The pH of the humus layer in depth less than 0.09 m is in the range of 5.6–6.5. The soil composition in the orchard is suitable for growing walnut trees. The climatic region is warm and very dry with a mild winter. The average annual air temperature reaches 9.8 °C. In January, the average temperature is below −2 °C, and in July, the temperature reaches an average of more than 20 °C. Table 1 shows the comparison of total precipitation and the mean air temperature during the studied period in 2019 with the long-term climatic normal of 1960–1991 [24]. The sum of the annual average global radiation was over 1.306 kWh m^−2^. The mean annual total precipitation is 523 mm [24]. The snow cover lasts less than 40 days a year. The Tomlain´s climatic indicator of irrigation averages more than 200 mm, indicating a lack of soil moisture [20]. 

The research took place in 2019, but in this study, we focused on the growing season. This period was chosen for its high water demand. The measured data on climatic conditions in the walnut tree orchard were provided by the owner of the orchard from a private meteorological station. The data for the Nové Zámky area were supplemented with data requested from Slovak Hydrometeorological Institute (SHMÚ) which were used for gap filling.

The water application doses in the orchard were delivered by a micro sprinkler irrigation system.

### 2.2. Experimental Design

**Figure 2:**
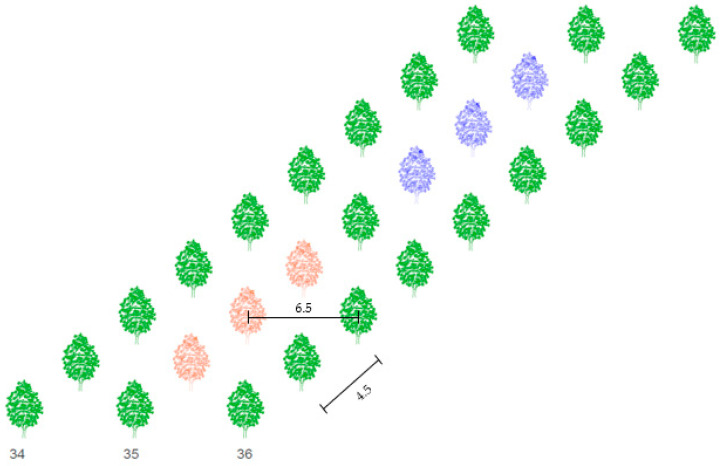
Scheme of spacing (m) between trees in the orchard and sample trees in row 35. Red color represents non-irrigated trees (treatment A), blue color represents irrigated trees (treatment B).

The area of the walnut tree orchard is 12.31 ha and trees are planted in 102 rows. The distance between sample trees and Nitra River is 500 meters. Spacing between rows is 6.5 m and spacing between trees in a row is 4.5 m (Figure 2). The height of sample trees reached 2.05 to 2.20 meters in 2019. The age of the sample trees in 2019 was 5 years. Trees in the row 35 where the meteorological station is placed were chosen for the study. Six trees were divided into groups A and B, each consisting of three tree individuals (Figure 2). Treatment A was non-irrigated, the water dose consisted only of natural rainfall. A dose of 30 mm was supplied in treatment B when the soil water potential rose to 80 kPa. The sap flow and dendrometric sensors have been installed on branches with diameters from 0.95 to 1.10 cm.

#### Crop

Walnut tree var. Chandler (*Juglans regia* L.) is a thermophilic tree with a massive crown. This species is widespread throughout the territory of Slovakia up to an altitude of 900 m. From a meteorological point of view, those are areas with frequent occurrence of late spring frosts [12]. Thus, it is necessary to closely monitor the development of tree growth. When over-irrigated, trees are prone to root rot, such as phytophthora root rot (*Phytophthora* spp.) [22,23]. 

In the conditions of West Slovakia, the emergence of leaf buds takes place from the beginning of April, and this period approximately lasts 20 up to 25 days. The beginning of flowering occurs from the end of April and lasts until the second half of May, seamlessly followed by the phenological stage of fruit ripening, which lasts until the beginning of October. The leaf fall period begins from about mid-October [25].

### 2.3. Field Measurements

#### 2.3.1. Soil Moisture

The soil moisture potential was measured by Irrometer® strain gauges (SR model, Riverside, CA, USA) that determine the electrical resistance of water in the soil. The sensors were in the effective root zone at locations that provided a representative picture of the soil water status in the depths of 0.2, 0.4, and 0.6 m. During the nut setting, surface irrigation was applied by micro sprinklers placed at a height of 0.3 m above the soil surface. Additionally, Irrometer™ tensiometers were used to measure the potential of soil water and its changes in the soil profile [26,27,28]. Values were measured in kilopascals [kPa]. 

#### 2.3.2. Sap Flow

SGEX-16 microsensors from Dynamax Inc. (Houston, TX, USA) were used to obtain sap flow values. The sensors were installed on the branches of walnut trees in both studied treatments—irrigated and non-irrigated.

**Figure 3:**
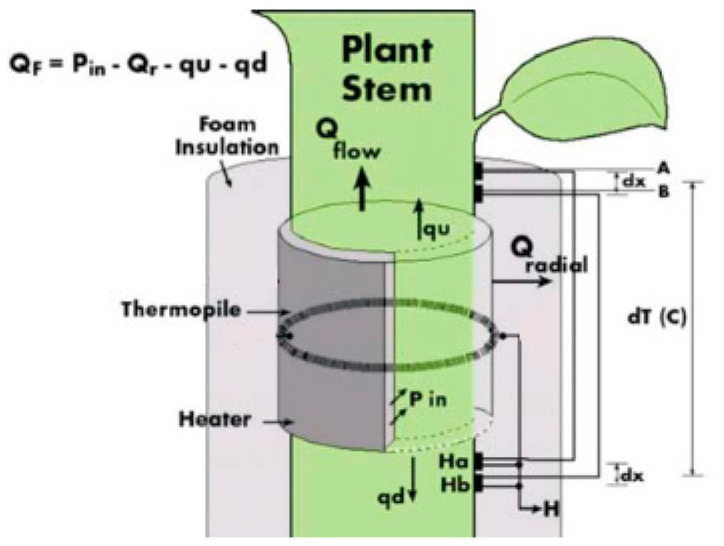
Sensor scheme for the energy balance calculation of sap flow (Dynamax Inc., 2020) [30]. Thermocouples A and B are placed above the heater and thermocouples Ha and Hb are placed below the heater to measure the difference between temperature above and below the heater.

Before the actual installation of the sensors, the installation sites were treated with natural oil to improve the thermal conductivity between the branch tissue and the thermocouple sensor. Each sensor was then coated with insulating foam to prevent heat losses from the thermocouple, which heats a small segment of the stem. The outer protective layer served as a reflective shield to protect the monitored spot from external environmental influences. The Dynagage Flow32-1K data logger (Dynamax Inc.) was used to collect data. The basic equation, thermodynamics, and calculation of sap flow are the same for all types of SG sensors of Dynamax company. Stem heat balance theory works on the principle of measuring the temperature difference above and below the heater [29]. This requires a steady-state and a constant supply of energy from the heating belt inside the sensor body [30,31]. An electric cell mounted on the stem continuously heats the surroundings of the sensor, and the heat removed during the sap flow is used to calculate the amount of sap flow in the stem in mass units per unit time. Continuous heating can also be replaced by short-term heat pulses that propagate along the stem. Based on their velocities, the amount of sap flow is then determined [19,32]. Figure 3 shows the individual components of the energy balance needed for the sap flow estimation.

Dynamax Inc. describes in detail the exact procedure for installing the sensors and dataloggers in their manual. The measured data from the sap flow devices were calculated according to the manual [29].

The energy balance of the heater (*P_in_*) is expressed by Ohm´s Law as the ratio of the input voltage (*V*) and the resistance of the heater (*R*):

*P_in_* = *V*^2^/*R* (W) (1)

*P_in_* can be expressed by the Equation (2):

*P_in_* = *Q_r_* + *Q_v_* + *Q_F_* (W) (2)

where *Q_r_* is the effect of the radius of the cylinder´s thermal conduction, *Q_V_* represents the vertical conduction components, *Q_F_* is the thermodynamic of a heated fluid in an insulated cylindrical section.

The conductive components—the flows above (*Q_u_*) and below (*Q_d_*) the heater—can be calculated by applying Fourier’s Law. By deriving *Q_u_* and *Q_d_*, *Q_v_* is calculated as

*Q_v_* = *K_st_* · A · (*BH* − *AH*)/(dx · 0.040) (—) (3)

where *K_st_* is the thermal conductivity of the stem (W m^−1^ °C^−1^). *K_st_* for a woody stem is equal to 0.422 W m^−1^ °C^−1^. *A* is the cross-sectional area of the stem (m^2^), *BH* and *AH* represent two differentially wired thermocouples both measuring the rise in sap temperature, and dx is the spacing between thermocouple junctions (m). The thermodynamics equation of a heated fluid in an insulated cylindrical section at a constant temperature (Equation (4)) indicates the source of the sheath conductivity (*K_sh_*) and the effects of the radius of the cylinder´s thermal conduction (*Q_r_*):

*Q_r_* = *K_sh_* · *CH* (—) (4)

where *CH* is the radial heat flux to the thermopile output. Both *K_sh_* and *CH* are sent to the datalogger, where the input to the datalogger is directly proportional to the temperature difference between the inner and outer layers of the cork substrate and, therefore, the radial heat transfer. 

Sap flow (*F*) is calculated by converting the residual heat (*P_in_*) to flow by dividing by an increase in the sap temperature and the heat capacity of water.

(5) $$F=\frac{P_{in}-Q_{v}-Q_{r}}{Cp\;\cdot \;dT}\;\left(g\cdot s^{-1}\right)\;$$

Since the sap is 99% water in the calculation, it is assumed that the heat capacity *Cp* is 4.186 J g^−1^ °C^−1^ [30,31,32,33,34,35]. The inputs to the equations described above are outlined in Figure 3.

*K_sh_* = *P*/*V* (—) (6)

If *F* ≈ 0, then *K_sh_* can be calculated as [34,35,36]:

To calculate sap flow, it is necessary to know the temperature increase of the sap (*dT*) in degrees Celsius. These values are measured in millivolts by the two thermocouples by averaging the *AH* and *BH* signals in degrees Celsius. To compute *dT*, the thermocouple temperature conversion constant (0.040 mV °C^−1^) is included in the equation:

(7) $$dT=\frac{AH+BH}{2}\times 0.040\;\left({}^{\circ}C\right)$$

#### 2.3.3. Dendrometric Measurements

The dendrometric changes were recorded with four Diameter Dendrometers Small (DD-S) instruments per sample (Ecomatik, Dachau/Munich, Germany). Dendrometers were installed in irrigated and non-irrigated groups on tree branches with a diameter from 0.95 to 1.10 cm. The device monitors changes in the diameter of the branch to an electrical signal with the resolution of 4.4 μm per volt in the range from 0 to 11,000 μm with an accuracy of ~2 μm [35]. The dendrometers were attached to the branches with rubber bands, which did not damage the tissue. The coefficient of thermal expansion of the sensor is ˂0.2 μm K^−1^. The values of electrical resistance were measured and recorded automatically every hour using a data logger DL 18. The obtained measured data were converted from ohms to μm by using the conversion Equation (8):

μm = raw data × 4400 (μm)(8)

### 2.4. Statistical Analyses

The Pearson coefficient of correlation (PCC) was used to determine the interrelationships between the following variables in both irrigated and non-irrigated treatment during budbreak and flowering periods:

- Sap flow and air temperature;
- Sap flow and soil moisture;
- Dendrometric changes and air temperature;
- Dendrometric changes and soil moisture;
- Sap flow and dendrometric changes.

PCC is a mathematical method that uses numerical expression to calculate the direction and degree of the relation between linear related quantitative or qualitative variables. The coefficient does not depend on the scale or origin.

## 3. Results

### 3.1. Budbreak Period

In this study, we focused on the budbreaking and flowering stages of walnut tree that are prone to water shortage—the budbreak phase from 28 March to 25 April 2019 and the flowering phase from 1 May to 2 June 2019.

**Figure 4:**
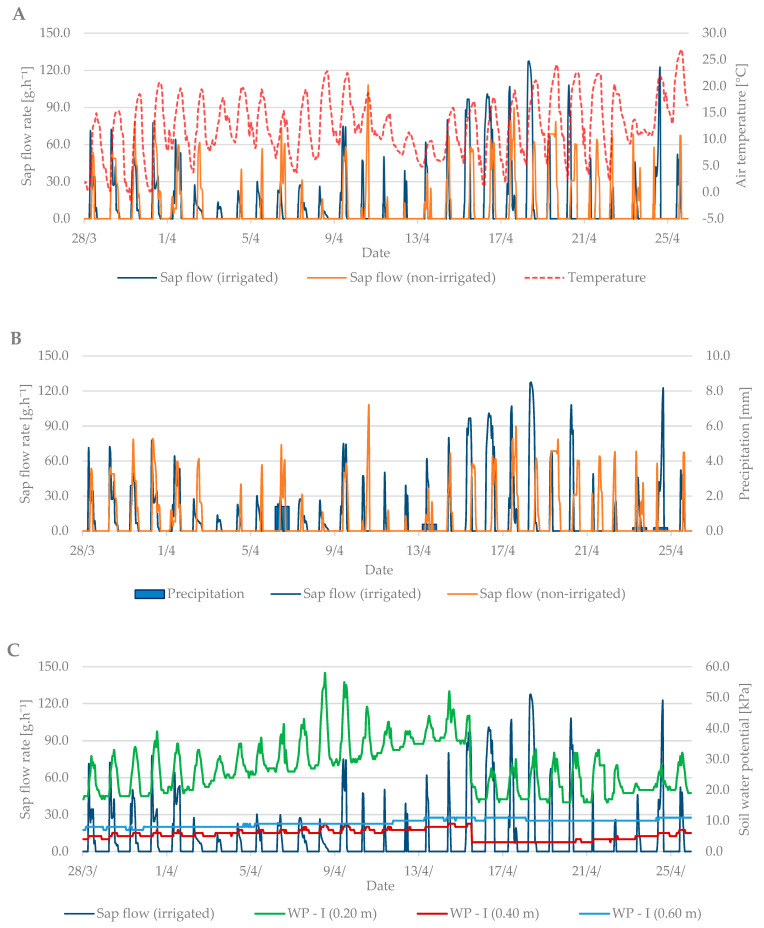

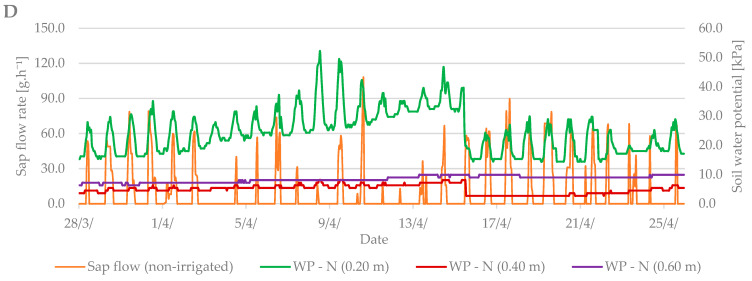
Diurnal course of sap flow rate during the budbreak period in 2019 in the irrigated and non-irrigated treatment depending on (**A**) air temperature; (**B**) amount of natural precipitation; (**C**) soil water potential values in the irrigated variant (WP − I) measured in the depth of 0.20, 0.40, and 0.60 m; and (**D**) soil water potential values in the non-irrigated variant (WP − N) measured in the depth of 0.20, 0.40, and 0.60 m.

Sap flow is affected by several factors such as: air temperature, precipitation, soil moisture, cumulative leaf area, and others. During the budbreak period in 2019, the average air temperature was 12.6 °C and it ranged from –1.8 °C to +27 °C (Figure 4A). The total precipitation for the first observed period was only 2.2 mm (Figure 4B). Soil moisture in kPa (Figure 4C) increased from the beginning of the measurements, indicating a lack of moisture in the soil profile at a depth of 0.2 m and the need for irrigation. After irrigation (15 April), the values fell by 20 kPa to 20–30 kPa when the soil, according to Table 2, was sufficiently moist.

Sap flow peaks when the air temperature is also at a maximum. The stomata open and the vapor is intensified; thus, the transpiration and flow of the sap are intensified. To cool the plant body sufficiently is the secondary effect of this process. At the beginning of the budbreak period, the sap flow rate of irrigated and non-irrigated trees was low (up to 80 g h^−1^). Total precipitation was insufficient, and the soil profile dried out. After irrigation, the difference between the irrigated and non-irrigated value of sap flow rate could be seen. In the irrigated treatment, the values ranged from 90 to 130 g·h^−1^. The sap flow rate in the non-irrigated variant remained at values below 90 g·h^−1^.

**Figure 5:**
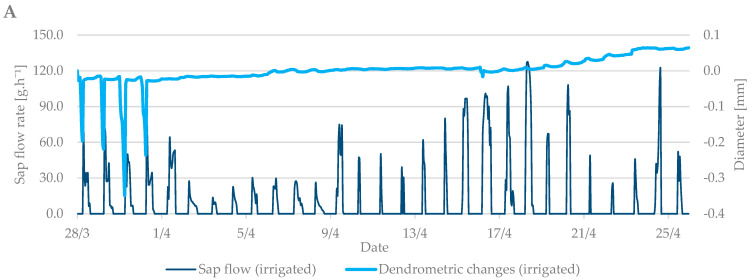

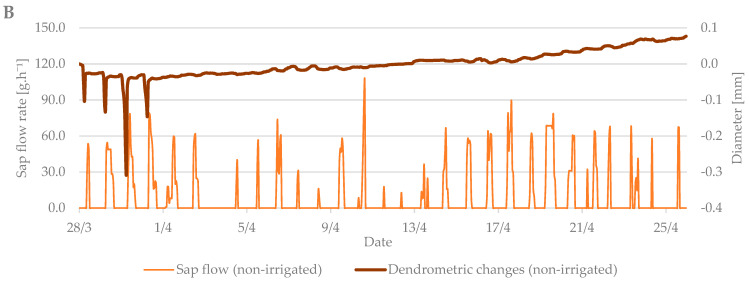
The shrinkage of branch diameter of irrigated (**A**) and non-irrigated (**B**) trees during the budbreak period in 2019 in comparison to sap flow rate.

The values from the dendrometric measurements were the highest during the evening and early morning hours, when the trees add water to the xylem cells and transpiration is minimal. On the contrary, the lowest values occurred at noon, when the sap flow is the highest and the air temperature also peaks. When comparing the dendrometric measurements, the radial growth of branches was not so intense during the bud break period relative to the radial growth of branches in flowering period (Figure 5). An increase in branch diameter was only 0.1 mm during the observed budbreak period. However, shrinkage occurred during periods with zero and negative air temperatures. The differences between the irrigated and non-irrigated treatments were minimal.

### 3.2. Flowering Period

**Figure 6:**
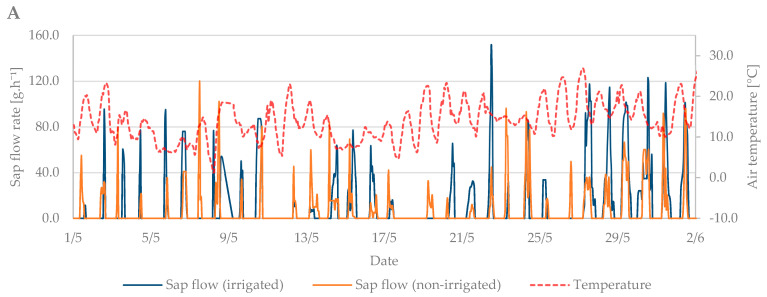

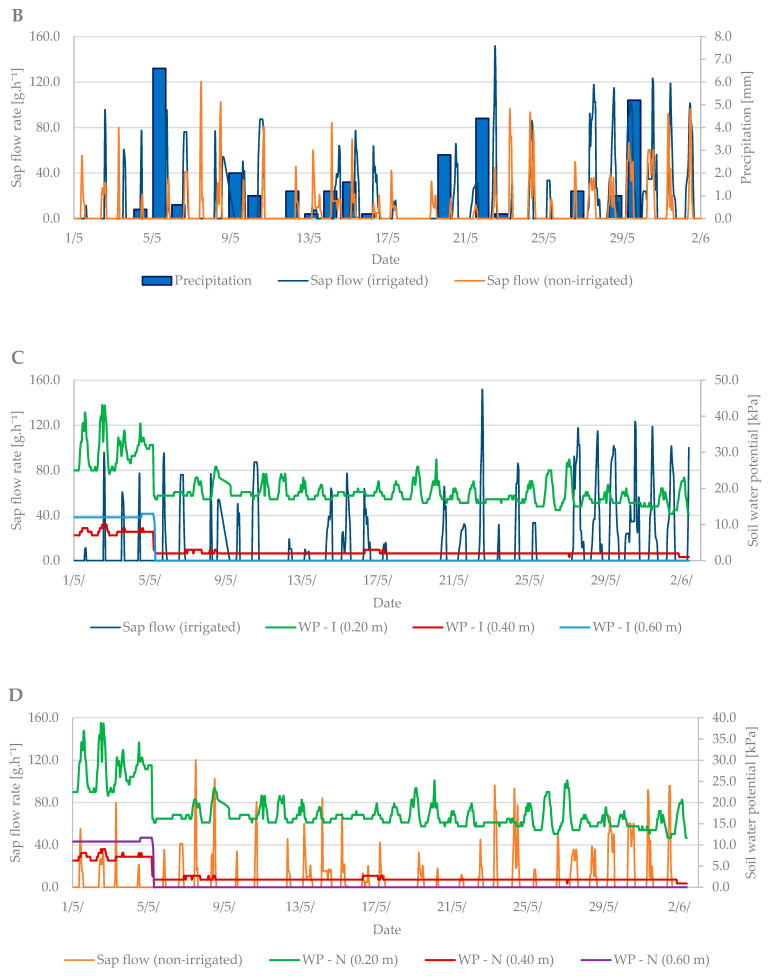
Diurnal course of sap flow rate during the flowering period in 2019 in the irrigated and non-irrigated treatment depending on (**A**) air temperature; (**B**) amount of natural precipitation; (**C**) soil water potential values in the irrigated variant (WP − I) measured in the depth of 0.20, 0.40, and 0.60 m; and (**D**) soil water potential values in the non-irrigated variant (WP − N) measured in the depth of 0.20, 0.40, and 0.60 m.

During the flowering period, the air temperature ranged from 1.3 °C to 27.5 °C. The mean air temperature was 14.4 °C (Figure 6A). Soil moisture at the beginning of this growth stage indicated the need for irrigation. However, from 4th May, rainfall was frequent, and soil moisture was sufficient without the need for irrigation. The total precipitation reached 28 mm, with 11 days with a total of over 1 mm and 16 days with a total of over 0.1 mm (Figure 6B).

The sap flow of the irrigated trees rose to 150 g·h^−1^ during the flowering period (Figure 6C). Overall, higher values of sap flow were observed during this period due to a greater number of leaves in the tree crown compared to the budbreaking period. The lowest sap flow values were observed when air temperature dropped from 15 May to 20 May. At that time, the trees did not decrease transpiration as intensively as at high air temperatures. Sap flow also increased during precipitation days compared with non-irrigated trees, which had significantly lower sap flow values (Figure 6C,D).

**Figure 7:**
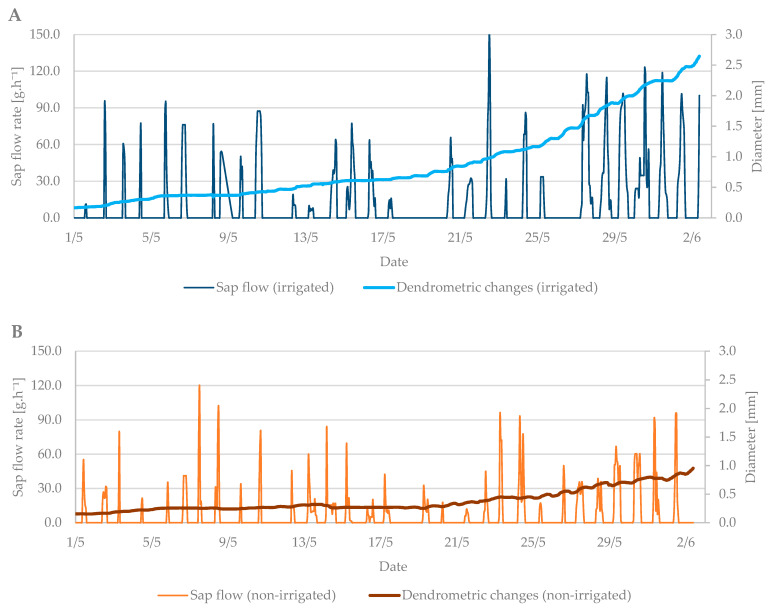
The shrinkage of stem diameter of the irrigated (**A**) and non-irrigated (**B**) trees during the flowering period in 2019 in comparison to the sap flow rate.

The dendrometric changes were more visible at this growth stage. The difference in branch diameter between irrigated and non-irrigated trees was more than 1.5 mm (Figure 7A,B). The larger the diameter of the branches was, the higher the sap flow in the irrigated treatment was as well. The branches in the non-irrigated treatment expanded their diameter only by 0.5 mm, and the sap flow remained at the level of previous measurements.

In general, the differences in sap flow rate between irrigated and non-irrigated treatments were observed in both studied growth stages (budbreak and flowering) when the trees needed enough moisture. The average difference was 40 g h^−1^. In the case of dendrometric measurements, the branch diameter of irrigated trees was larger by up to 1.5 mm compared to non-irrigated trees; however, these differences were observed only during the flowering stage.

### 3.3. Relation between Sap Flow, Dendrometric Changes and Selected Properties of Environment (Air Temperature and Soil Moisture)

In general, a significant correlation was observed between sap flow (irrigated and non-irrigated treatment), air temperature (T), and soil moisture (w) at budbreak period (Table 2). The only exceptions were the relationships of sap flow with soil moisture in the depth of 0.20 and 0.60 m in the non-irrigated treatment. On the contrary, no significant relationships with sap flow were found at the flowering growth stage. Regression analyses overall showed significant correlations between dendrometric changes and air temperature and soil moisture in both treatments and during both studied growth stages (budbreak and flowering). However, the relationships between dendrometric changes and soil moisture at the depth of 0.20 and 0.40 m in the irrigated treatment were insignificant at budbreak stage. In the end, sap flow measurements significantly correlated with dendrometric changes in both treatments and studied periods (Table 3).

## 4. Discussion

The projected climate scenario for Slovakia assumes an increase in the need for additional irrigation in the Danube lowland as a result of climate change in the coming years [36]. In 2019, the Danube Plain region was characterized by very low precipitation in the spring. During the studied period, precipitation was 36.6% lower than the long-term average for this area (Table 1). The reaction of the examined trees to the deteriorated water status was due to the lack of natural precipitation in the initial phenological stages. There is a relationship between soil moisture and sap flow: a drop in precipitation causes a gradual decrease in transpiration and, thus, a reduction in sap flow [37], whereby the tree growth begins to show signs of water deficit.

The physiological changes of plants during a diurnal cycle are based on performance between transpiration and the day and night physiological cycle of trees [38]. Diurnal changes in sap flow rate, which are significantly affected by the intensity of solar radiation, air temperature, and changes in soil moisture, were also verified by Chang et al. [39]. During the budbreaking period, they noted a difference in sap flow in trees of Qinghai spruce (*Picea crassifolia*). The difference in sap flow value was different in different species during the budbreak period. The walnut develops leaves, but the spruce has permanent needles. Our results are in line with their observations that there is a significant relationship between soil moisture and sap flow velocity rate in the irrigated treatment. This effect is caused by a relationship between the rate of sap moving from the roots to the leaves, through the stem, and transpiration process [40]. The canopy structure as well as its area and shape are important aspects in determining sap rate during the growing season [41]. The sap flow rate is minimal in the period before bud break. It is caused by the minimal transpiration because of unfold leaves. The sap rate and unfolding leaves are in a linear relationship in the period from full bud opening to full bud unfolding [42]. In the non-irrigated treatment, the sap flow peak was up to 40 g·h^−1^ lower than in the irrigated treatment, which reached up to 130 g·h^−1^. Zha et al. [43] interpret precipitation as the most important source of soil moisture; thus, walnut trees require a large volume of water supply during periods of low precipitation when the sap flow movement in the xylem tissue is slower. Sap flow during the budding period began to increase in the irrigated variant as a result of an increase in the volume of water in the tissue, and its value subsequently slowly decreased [44]. The highest daily air temperature of 19.2 °C was reached at 4 p.m. The non-irrigated treatment reached its maximum value in the same hour. During the budbreaking phase, the total natural precipitation was only 2.2 mm. The radial increase was recorded especially in the early morning and late evening hours when the transpiration value was lower due to lower air temperatures. On the contrary, the greatest shrinkage of the tissues was recorded during the noon hours in both irrigation treatments. During the budbreaking period, a maximum radial increment of only 0.1 mm was measured. At this stage of development, the diameter of the branch shrunk due to the low night temperatures at this time of year.

Close relationships between air temperature and the course of cambial activity, as well as the amount of xylem produced, were observed in previous studies [44,45,46]. Decreased cambium activity due to drought can be quickly restored by increasing the water content in the available soil profile [47]. Change in the volume of water in the tissues of trees is also one of the factors that contribute significantly to the course of dendrometric changes in the tissues during the daily cycle of trees [48,49]. The physiological changes of plants during the day are divided into four phases depending on the value of transpiration, which changes under the influence of sunlight and temperature and affects the values of sap flow and radial changes in the tree trunk. Between separate phases is indicated time lag between transpiration and sap flow. The first phase takes place at night when the transpiration value is lowest; the plant rehydrates, and the diameter of the stem increases with a larger volume of water in the plant tissue. The sap flow value is the lowest at this stage. The second phase overlaps the delay between an increase in sap flow after sunrise. The third phase continues by reducing the strain to the maximum value of sap flow. In the late afternoon, the sap flow rate starts to decrease, and this continues during the night until sunrise [50,51].

During the flowering period, when the leaves are already fully developed, transpiration and, thus, the sap flow naturally increase; in the irrigated treatment, the sap flow reached a maximum value of 150 g·h^−1^, and in the non-irrigated treatment, values were smaller by up to 50 g·h^−1^ (Figure 6). In the early phenological stages of walnut, the availability of soil water at different depths of the soil profile also has a great influence. Liu et al. [52] studied the water consumption of walnut and described the changes in the depth of soil water abstraction using roots during different phenological stages. Their work showed that in the period of budbreaking, the largest share of water is taken from the upper 0–0.20 m layer of a soil profile, and at the onset of flowering, the share of water extracted from greater depths increases. 

As a response of the plants to the low precipitation and subsequent low soil moisture, the sap flow rate in xylem vessels is decreasing [53]. Our research showed the impact of irrigation on the selected biophysical properties (dendrometric changes and sap flow rate) of walnut trees. The effect of irrigation on the sap flow is indirect based on the relation between soil moisture and irrigation dose [54,55], which is also the case in our study.

The combination of methods of monitoring the sap flow rate and monitoring the dendrometric changes can be used as a basis for streamlining water use in arid areas [49]. The water storage of the stand can be controlled by irrigation systems. Dang et al. [47] monitored the effects of drought on the values of radial enlargement of the Mongolian Scots Pine strain (*Pinus sylvestris* var. *mongolica* Litv.) for five years. Their observations showed a reduction in radial gain of up to 31% because of drought conditions. They observed that even after drought, the cambium growth did not recover to 100%, and in the case of some species, the recovery lasted for more than two years.

## 5. Conclusions

In this study, we described biological changes in individuals of walnut tree (*Juglans regia* L.) due to environmental conditions—air temperature, soil moisture, and additional irrigation. The results show that monitoring the physiological changes of trees by measuring sap flow and dendrometric changes allows us to monitor the water regime of trees on a daily basis. Additional irrigation in dry days during the first phenological phase (budbreak) contributed to more intensive trunk growth. The difference was 1.5 times higher compared to trees in non-irrigated treatment under natural precipitation regime. In terms of agricultural production and ongoing climate change, it is necessary to continue research directed toward the impact of irrigation on physiological changes in plants. Continuation of this research and analysis of the data obtained in the coming years will allow us to evaluate the effects of the environment on fruit trees in the long term.

## Figures and Tables

**Table 1:** Precipitation totals and mean monthly air temperature in Nové Zámky in the growing season 2019 in comparison with climatic normal (1960–1991) [24].

Month	Precipitation			Air Temperature		
	Total (mm)	% of Normal	Description	Average (°C)	Deviation from Normal (°C)	Description
March	17.7	65.56	dry	8.3	+5.3	warm
April	2.8	3.11	extremely dry	13.2	+2.5	very warm
May	29.8	53.21	dry	14.0	+1.6	warm
June	9.2	15.08	extremely dry	23.9	+5.3	extremely warm
July	50.7	99.41	normal	23.4	+3.2	extremely warm
August	7.4	12.76	extremely dry	22.9	+3.4	extremely warm
September	5.8	14.87	extremely dry	15.8	+0.3	normal
October	17.7	55.31	dry	12.5	+2.3	warm

**Table 2:** Pearson correlation coefficient between sap flow, air temperature, and soil moisture, and dendrometric changes with air temperature and soil moisture in the irrigated and non-irrigated treatments.

		Budbreak Period				Flowering Period			
	Treatment	T (°C)	w (mm)			T (°C)	w (mm)		
			0.20	0.40	0.60		0.20	0.40	0.60
Sap flow	Irrigated	0.27 ***	0.25 ***	0.34 ***	0.50 ***	0.06	0.05	0.03	0.05
	Non-irrigated	0.21 **	0.02	0.18 *	0.14	0.89	0.01	0.001	0.004
D.C.	Irrigated	0.35 ***	0.01	0.42	0.58 ***	0.42 ***	0.47 ***	0.43 ***	0.40 ***
	Non-irrigated	0.28 ***	0.13 **	0.21 ***	0.72 ***	0.38 ***	0.47 ***	0.42 ***	0.39 ***

Notes: T—air temperature; w—soil moisture. * *p* < 0.05; ** *p* < 0.01; *** *p* < 0.001, D.C.—dendrometric changes.

**Table 3:** Pearson correlation coefficient between sap flow and dendrometric changes in the irrigated and non-irrigated treatment.

	Treatment	Budbreak Period	Flowering Period
		Dendrometric Changes	
Sap flow	Irrigated	0.24 **	0.17 **
	Non-irrigated	0.16 *	0.23 ***

(* *p* < 0.05; ** *p* < 0.01; *** *p* < 0.001).

## Data Availability

The data generated during and/ or analyzed during the current study are available from the corresponding author on reasonable request.
